# Hoof Pressure Distribution Pattern of Blue Sheep During Walking on Different Slopes: A Subject-Specific Analysis

**DOI:** 10.3389/fvets.2021.633509

**Published:** 2021-04-09

**Authors:** Xiangyu Liu, Hailin Kui, Zhihui Qian, Lei Ren

**Affiliations:** ^1^College of Transportation, Jilin University, Changchun, China; ^2^College of Biological and Agricultural Engineering, Jilin University, Changchun, China; ^3^Key Laboratory of Bionic Engineering, Jilin University, Changchun, China; ^4^School of Mechanical, Aerospace and Civil Engineering, University of Manchester, Manchester, United Kingdom

**Keywords:** blue sheep, different slopes, hoof, pressure plate, vertical force distribution

## Abstract

The purpose of this study was to quantitatively assess the vertical force distribution (VFD) of subject-specific healthy blue sheep while walking on different slopes using a pressure-sensing walkway. The blue sheep was trained to walk over the pressure-sensing walkway by choosing a comfortable walking speed, and the slope angle increased from 0° to 25°. The sheep's hooves were divided into four quadrants, namely, the cranio-lateral, cranio-medial, caudo-lateral, and caudo-medial quadrants, to investigate the VFD of the peak vertical force (PVF), vertical impulse (VI) and occurrence time of the PVF during the stance phase (TPVF). This study demonstrates that the main stressed quadrant of the front hoof changes from the caudo-medial quadrant to the cranio-medial quadrant with increasing slope. The main stressed quadrant of the rear hoof is the cranio-medial quadrant and does not change with the increasing slope. For all the slopes, the vertical force shifted from the lateral quadrant to the medial quadrant and from the caudal quadrant to the cranial quadrant. All the results obtained in the study suggest the feasibility of detecting gait changes in blue sheep, which has potential for the diagnosis of lower limb musculoskeletal diseases in quadrupeds.

## Introduction

The effective diagnosis of skeletal and musculoskeletal diseases of terrestrial animals is of great importance, potentially benefiting the health of various animals (1–3). Radiography is a widely used method for the diagnosis of animal bone diseases. However, this method has the potential to cause harm to animals, cannot identify early disease and is not sensitive to pathological changes (4). Gait is a fundamental function of animals and involves all movement, such as walking, running, jumping and climbing (5). Lower limb musculoskeletal diseases in animals exhibit obvious manifestations during exercise, such as lameness (5), and dogs may change their posture during walking due to pain (6). Therefore, gait analysis of animal models can be an objective method by which to document limb function or to analyze changes that are related to limb musculoskeletal disease. Since this method is non-invasive and does not influence experimental conditions, gait measurement has been used clinically as an indicator to guide the course of treatment and evaluate efficacy (7, 8).

Vertical force distribution (VFD) is often used to evaluate the gait of humans and animals (6, 9, 10) and allows for the evaluation of a variety of foot diseases in humans (9–12) and descriptions of limb function in animals (2, 8, 13, 14). In previous animal gait studies, vertical force was commonly measured with pressure plates (2, 10, 15, 16). Pressure sensing systems have been presented as a viable alternative to traditional force platforms, and in animal studies, they have the advantage of be able to record multiple foot pressures during walking (17).

Oosterlinck et al. used a pressure plate to study the vertical force of horse hooves and found that the symmetry of the vertical force of the right and left hooves was higher for walking than for trotting (15). Besancon et al. compared the VFDs of greyhounds and Labrador retrievers during walking and found significant differences between the breeds (13). Romans et al. used a pressure plate to analyze the effect of bilateral onychectomy on the VFD of cat claws, and the results showed no significant difference in the peak vertical force (PVF) between cats that had and had not undergone bilateral onychectomy (18). Rifkin et al. analyzed the difference in the VFD among goat limbs (3). Results showed that no difference between the left and right limbs, and there were significant differences between the front limbs and hind limbs. Maximum force and maximum peak pressure of the front limbs are significantly higher than those of the hind limbs. In a recent study, pressure-sensing walkways were used as a biometric tool for gait and force to quantitatively evaluate the gait characteristics and vertical pressure of healthy goats (1, 3). In that study, the complete goat hoof print was analyzed as a whole. Stadig et al. divided the paw prints into four equally sized areas when analyzing the VFD in the cat's paw. The results showed that the main weight transferred during a strike is from the caudal quadrants toward the cranio-medial quadrant of the paw (2). Additionally, Schwarz et al. divided paw prints into four quadrants to study the VFD in dogs, and the results showed that the PVF in the cranial quadrants was higher than that in the caudal quadrants (16). The above two studies showed that more detailed VFD information could be obtained by partitioning paw prints. Therefore, quadrant division of paw prints can be more accurate than complete prints for determining whether an animal's lower limbs exhibit musculoskeletal diseases.

To our knowledge, the subjects in previous studies all walked on flat ground. These studies mostly focused on biomechanical properties and diagnostic methods for lower limb musculoskeletal disease in animals walking on flat ground. However, studies have shown that the musculoskeletal system of some quadrupeds, such as blue sheep, which live in mountainous areas, is often influenced by harsh living environments (19, 20). For animals such as the blue sheep, climbing, and climbing exercises are more likely to cause injury to the animal's lower limb musculoskeletal system. However, some injuries during climbing are not easily detected when walking on flat ground. Therefore, how to make a quick and non-invasive preliminary diagnosis of lower limb musculoskeletal diseases in hoofed animals while climbing in the wild is still challenging. Therefore, it is necessary to incorporate climbing when studying the VFD of blue sheep. Blue sheep are even-toed hooved animals, and the hoof toes can move freely during the process of movement. Therefore, it is also necessary to divide the hoof marks into different regions when studying the VFD of these hooves.

The purpose of this study was to analyze the biomechanical characteristic parameters of blue sheep hooves by dividing them into four quadrants. The subject-specific blue sheep was trained to walk on flat ground and different slope angles obtain the biomechanical characteristic parameters, which may provide a reference method for the future study of lower limb musculoskeletal disease diagnosis in blue sheep through a pressure-sensing system.

## Materials and Methods

### Ethical Statement

This study was approved by Changchun Zoological and Botanical Garden, and experiments were carried out under the guidance of relevant personnel.

### Animals

Blue sheep are under second-class protection in China and have been included in the International Union for Conservation of Nature's Red List of Threatened Species; hunting and trading these animals are prohibited. The blue sheep subject was supplied by a local zoo (Changchun, China) and was 4 years old, weighed 38.2 kg, and had no history of musculoskeletal disease. Because it is difficult to recruit numbers of blue sheep to participate this measurement. The blue sheep that employed in the study, is considered to be less differentiated after a long period of observation of the state of movement of the blue sheep group as it walks on the flat ground. To some extent, it can be thought that a single blue sheep represents part of the characteristics of the population, similar to the sample acquisition method used by Lewinson et al. (21) for the s mountain goat climbing dynamics description experiment. In order to reduce the experimental error, we carried out a large number of experiments on a single sample in order to obtain more reliable experimental data.

### Experimental Scheme Design

**Figure 1:**
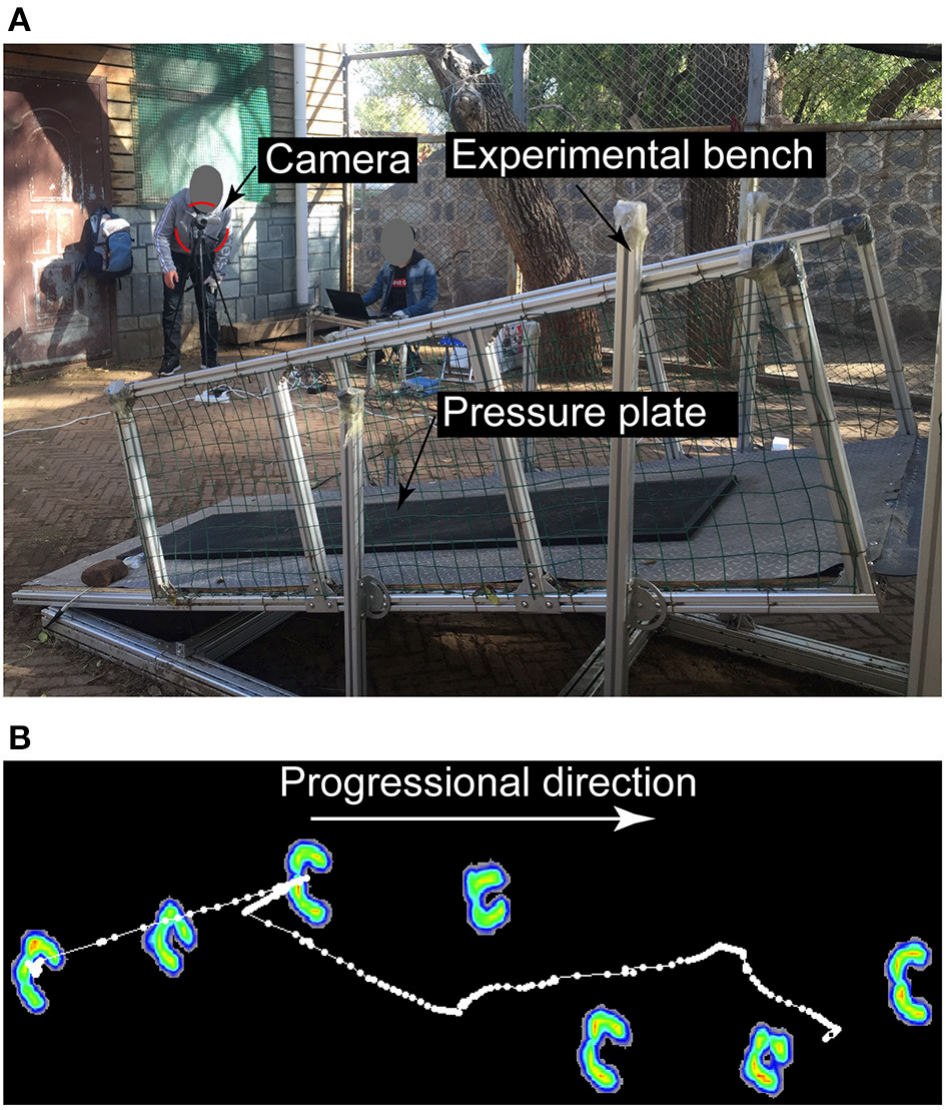
**(A)** VFD acquisition system. This includes a pressure plate for collecting the VFD, an experimental bench with the function of changing the slope angle and a camera for recording the experimental process. **(B)** Image of the pressure plate recordings of the forces acting on the hooves over time.

An experimental platform was designed to change the angle of the ramp; the angle increased linearly from 0° to 25° at intervals of 5° during the experiment (see Figure 1A). Prior to measurement, the platform was installed and left 1 month for the blue sheep subject to adapt to it. During the month, the angle of the ramp was changed every 5 days to allow the sheep to fully adapt to each slope angle. Before the start of the experiment for each slope angle, the breeder used food to induce the subject to walk freely around the test site and then pass the ramp. The subject walked several times on the ramp to set its own comfortable speed. The results of the experiment were accepted only if the subject did not swing its head from side to side during walking. All experiments for the same slope were completed on the same day, and the subject walked a minimum of ten times over the pressure plate to ensure five valid motion data points (*n* = 5).

### Pressure Data Collection

Hoof pressure data were collected using a 2,093 × 469 mm pressure plate (RSscan International) situated in the middle of a 2.5 m ramp. Sensors with a length of 7.62 × 5.08 mm were embedded in the pressure plate, and the sampling frequency was 126 Hz. A camera (Sony HDR-CX680) was used to record the motion to calculate the walking speed (3) (Figure 1A).

### Effective Sample Selection

It has been reported that velocity is an important factor that influences the ground reaction force and the VFD (12, 22). The velocity of the blue sheep is represented by the stride velocity of the right forelimb. The stride velocity is the stride length divided by the stride time. The stride length is defined as the distance measured parallel to the line of progression between the heel points of two consecutive footfalls of the right forelimb. The stride time is the elapsed time between the first contacts of the two consecutive footfalls of the right forelimb (3). The stride length is the ratio of the advance distance in the video to the known length of the pressure plate. To reduce the influence of speed on this study, samples with similar speeds were defined as effective samples during the screening of experimental data in our study.

The main error source in this study is that the head position of the blue sheep subject was not centered during walking. Previous studies have demonstrated that the PVF of the front limb is significantly higher for the side to which the head is positioned (2, 23). In this study, we used a camera system to record the walking posture of the blue sheep. Samples with head centring were considered usable. This method does not guarantee that the head of each sample is exactly in the center, but it can minimize the error.

### Data Analysis and Outcome Parameters

As shown in Figure 1B, the pressure plate measures the VFD on the bottom of the blue sheep's hooves. The hoof prints were manually identified with the help of special software (Footscan 7 gait 2nd generation), and the experimental data were analyzed. To analyze the VFD, the hoof prints were divided into quadrants (cranio-lateral, cranio-medial, caudo-lateral, and caudo-medial quadrants) (16).

**Figure 2:**
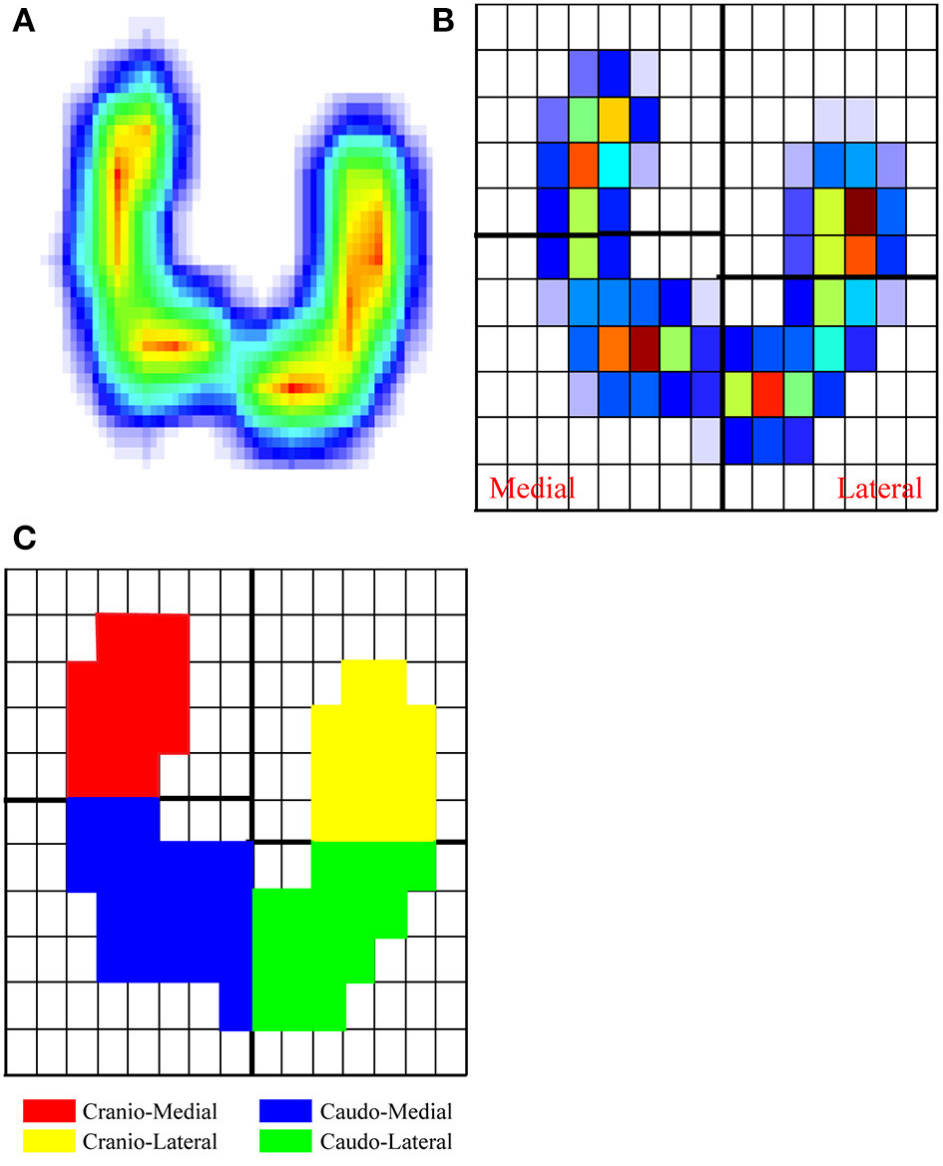
Procedure to divide the hooves (example of the right front hoof) into four equally sized quadrants. **(A)** Hoof print generated by the Footscan system, showing the hoof pressure distribution during the stance phase. The pressure distribution is color-coded from blue (low pressure) to red (high pressure). **(B)** Pressure distribution of the sensor embedded in the force plate. **(C)** The hoof is divided into four quadrants.

The quadrants were defined according to the following method. Blue sheep have split hooves; that is, their hooves are split into two toes. The VFD was obtained by the Footscan system. Figure 2A shows the VFD of sheep hooves, where the two regions correspond to the two toes. Taking the right front hoof as an example, we define the left region (medial region) as the inner quadrant and the right region (lateral region) as the outer quadrant. The relationship between the vertical force on the right front hoof and the sensor position is shown in Figure 2B, and each box indicates a sensor. Along the direction of travel, the sensors in the medial quadrant and the lateral quadrant are evenly divided into cranial and caudal quadrants from front to rear. Through the above method, the hoof prints are divided into the four cranio-lateral, cranio-medial, caudo-lateral, and caudo-medial quadrants (see Figure 2C).

The vertical force distribution data can be used to obtain the force time curves in each quadrant (see Supplementary Figure 1). Based on the measurement data, five parameters were analyzed and compared, including the PVF, vertical impulse (VI), occurrence time of the PVF during the stance phase (TPVF), start time of the vertical force during the stance phase (TSVF), and end time of vertical force during the stance phase (TEVF).

### Statistical Analysis

Statistically significant differences in the parameters (PVF, VI, TSVF, TPVF, and TEVF) between different quadrants of the same hoof and the parameters (PVF, VI, TSVF, TPVF, and TEVF) between different hooves in the same quadrant were identified by using one-way analysis of variance (ANOVA). The significance level for the ANOVA was set to 0.05 (2).

## Results

### Sample Validity

The walking velocity of the blue sheep subject for different slopes was 1.11 ± 0.08 m/s (0°), 1.12 ± 0.12 m/s (5°), 1.05 ± 0.02 m/s (10°), 0.78 ± 0.03 m/s (15°), 0.99 ± 0.07 m/s (20°), 1.31 ± 0.11 m/s (25°). For each sample, a speed error < 10%, was within an acceptable range, and the sample was considered a qualified sample.

The evaluated parameters were analyzed according to the following comparisons: (a) a comparison of each quadrant to the other quadrants of the same hoof, (b) a comparison of each quadrant to the corresponding quadrants of the other hooves, and (c) a comparison of the PVF and VI of each quadrant for the different slopes.

### Distribution Patterns of Blue Sheep Walking on the Ground

When the subject walked on flat ground, with the exception of the left cranio-lateral quadrants, the front hooves showed a higher PVF than the rear hooves see Table 1. In general, with the exception of the left cranio-medial quadrants, the front hooves showed a higher VI than the rear hooves (see Table 2).

**Table 1:** PVF in newtons (N).

**Gradient**		**Cranio-Medial**	**Caudo-Medial**	**Cranio-Lateral**	**Caudo-Lateral**
0	RF	44.26 ± 10.98	58.59 ± 15.52^a,b^	40.16 ± 4.60^1,d,e^	74.13 ± 14.00^1,f^
	RR	34.36 ± 6.31	28.85 ± 11.73^2,a^	30.85 ± 5.63^3,d^	55.45 ± 5.37^2,3,f^
	LF	50.42 ± 12.15^4,5^	79.53 ± 11.49^4,b,c^	31.17 ± 5.85^5,6,e^	78.16 ± 12.38^6,g^
	LR	42.03 ± 4.08	33.20 ± 3.86^7,c^	37.22 ± 6.37	45.37 ± 13.04^7,g^
5	RF	32.42 ± 6.87	45.00 ± 7.02^a^	27.68 ± 10.02	36.30 ± 2.01
	RR	27.57 ± 6.06^1^	14.72 ± 8.48^1,2,a^	27.13 ± 2.79	30.97 ± 4.22^2^
	LF	24.15 ± 6.63^3^	43.13 ± 10.89^3,b^	20.08 ± 4.60	32.10 ± 13.04
	LR	26.70 ± 3.76^4,5^	12.26 ± 2.28^4,6,b^	17.10 ± 4.33^5^	28.57 ± 6.73^6^
10	RF	27.80 ± 5.20^1^	38.26 ± 7.96^1,a^	20.87 ± 7.17^2^	31.56 ± 4.23^2^
	RR	32.20 ± 10.74	26.57 ± 3.84	26.57 ± 2.61	31.28 ± 6.07
	LF	35.53 ± 4.97^3^	57.92 ± 11.17^3,4,a,b^	25.70 ± 2.62^5^	43.77 ± 6.55^4,5,c^
	LR	33.23 ± 2.13	23.80 ± 6.49^b^	26.06 ± 5.49	29.56 ± 10.30^c^
15	RF	45.74 ± 8.91^1,a^	45.77 ± 12.26^b^	26.06 ± 6.28^1,2,e^	49.82 ± 14.19^2,f^
	RR	35.54 ± 9.77	29.89 ± 3.78^b,c^	32.32 ± 2.99	36.46 ± 6.63^f^
	LF	26.96 ± 5.18^3,a^	51.94 ± 11.76^3,d^	33.88 ± 3.38^4,e^	41.33 ± 8.11^g,4^
	LR	37.12 ± 6.75^5,6^	17.28 ± 4.83^5,7,c,d^	28.89 ± 6.55^6^	29.19 ± 3.67^7,g^
20	RF	33.66 ± 5.63^1,2^	26.24 ± 3.74^1,b^	25.9 ± 5.48^2^	28.55 ± 4.38^d^
	RR	31.79 ± 2.32^a^	26.14 ± 6.71	30.27 ± 5.32	29.27 ± 11.59
	LF	38.31 ± 7.81^3,4^	53.63 ± 5.88^3,6,b,c^	23.05 ± 4.49^4,5^	40.49 ± 5.39^5,6,d,e^
	LR	42.04 ± 6.78^7,8,a^	22.05 ± 4.70^7,9,c^	29.04 ± 2.33^8^	28.24 ± 2.61^9,e^
25	RF	41.56 ± 7.34^1^	14.44 ± 9.84^1,a^	40.39 ± 8.83^2,c^	9.55 ± 4.25^2,d^
	RR	37.82 ± 8.63^3^	18.30 ± 5.32^3,4^	35.61 ± 3.69	38.15 ± 9.43^4,d,e^
	LF	40.54 ± 4.89^5,6^	26.15 ± 10.54^5,7,a,b^	29.07 ± 4.94^6,8,c^	15.93 ± 2.58^7,8^
	LR	41.93 ± 4.74^9,10^	10.45 ± 3.04^9,11,b^	33.94 ± 3.66^10,12^	20.39 ± 4.52^11,12,e^

*RF, right front hoof; RR, right rear hoof; LF, left front hoof; LR, left rear hoof*.

*Mean (n = 5) ± standard deviation. The same letter denotes values in the same column with statistically significant differences (comparison of quadrants in the same hoof), and the same number denotes values in the same row with statistically significant differences (comparison of equal quadrants of each hoof)*.

**Table 2:** VI in newton seconds (Ns).

**Gradient**		**Cranio-Medial**	**Caudo-Medial**	**Cranio-Lateral**	**Caudo-Lateral**
0	RF	18.89 ± 6.67	27.45 ± 7.29^1,a,b^	22.01 ± 3.39^d,e^	32.77 ± 8.25^1^
	RR	16.72 ± 3.60	16.69 ± 5.99^2,a^	16.89 ± 4.58^3,d^	23.89 ± 5.49^2,3,f^
	LF	19.22 ± 6.64^4^	37.84 ± 3.05^4,b,c^	16.12 ± 2.59^5,e^	33.43 ± 8.08^5,g^
	LR	20.67 ± 3.39^6,7^	14.54 ± 1.75^6,c^	15.46 ± 0.95^7^	11.50 ± 0.32^f,g^
5	RF	9.86 ± 3.00^1^	16.58 ± 3.24^1,2,a^	13.83 ± 2.18^c^	11.33 ± 1.72^2^
	RR	10.84 ± 2.31	6.71 ± 2.65^a^	11.28 ± 2.17^d^	8.98 ± 1.00
	LF	6.73 ± 2.23^3^	17.12 ± 4.84^3,4,b^	9.15 ± 1.88^c^	10.03 ± 3.89^4^
	LR	10.28 ± 2.89^5,6^	4.61 ± 7.79^5,b^	5.99 ± 1.53^6,d^	7.30 ± 1.03
10	RF	8.32 ± 2.26^1^	13.78 ± 4.40^1^	10.41 ± 3.97	8.91 ± 1.45
	RR	11.60 ± 5.96	8.85 ± 1.61	9.71 ± 1.80	8.64 ± 3.37
	LF	8.69 ± 1.26^2^	18.82 ± 2.22^2,3,a^	9.03 ± 1.61	8.62 ± 1.10^3^
	LR	12.69 ± 1.94^4,5^	8.38 ± 2.43^4,a^	8.94 ± 2.10^5^	7.57 ± 2.22
15	RF	10.98 ± 2.32^a^	11.81 ± 2.80	7.56 ± 2.56	9.54 ± 3.65
	RR	10.05 ± 1.71	9.31 ± 1.88^c^	10.48 ± 3.22	9.42 ± 2.87
	LF	6.96 ± 1.89^1,a,b^	15.26 ± 4.61^1,2,d^	9.22 ± 2.43	7.59 ± 3.09^2^
	LR	12.52 ± 3.49^3,4,b^	5.19 ± 2.18^3,c,d^	8.67 ± 2.01^4^	7.21 ± 2.49
20	RF	9.22 ± 2.67	7.40 ± 2.02^b^	8.83 ± 1.56^1,d^	5.29 ± 1.02^1,e^
	RR	11.50 ± 1.44	9.05 ± 2.94	10.86 ± 2.02	9.82 ± 3.40^e,f^
	LF	10.76 ± 2.82^2,3,a^	15.89 ± 0.69^2,4,b,c^	7.76 ± 0.95^3,d^	6.63 ± 1.21^4^
	LR	14.30 ± 2.14^5,6,a^	6.40 ± 2.06^5,c^	9.50 ± 0.72^6,7^	6.63 ± 1.21^7,f^
25	RF	11.49 ± 1.59^1^	3.67 ± 2.42^1^	11.28 ± 2.27^2^	1.41 ± 1.10^2,d^
	RR	13.75 ± 3.02^3^	6.13 ± 2.69^3,4,b^	13.33 ± 2.14	12.13 ± 2.89^4,d,e^
	LF	10.59 ± 1.37^5,a^	7.17 ± 3.25^5,6,c^	9.60 ± 2.07^7^	2.84 ± 1.54^6,7^
	LR	14.29 ± 2.14^8,9,a^	2.24 ± 0.63^8,b,c^	11.97 ± 1.76^9,10^	3.85 ± 0.84^10,e^

*RF, right front hoof; RR, right rear hoof; LF, left front hoof; LR, left rear hoof*.

*Mean (n = 5) ± standard deviation. The same letter denotes values in the same column with statistically significant differences (comparison of quadrants in the same hoof), and the same number denotes values in the same row with statistically significant differences (comparison of equal quadrants of each hoof)*.

For the front hooves, the caudo-medial quadrants showed a higher PVF and VI than the cranio-medial quadrants (see Tables 1, 2), and the caudo-lateral quadrants showed a higher PVF and VI than the cranio-lateral quadrants (see Tables 1, 2). In contrast, for the rear hooves, the cranio-medial quadrants showed a higher PVF and VI than the caudo-medial quadrants (see Tables 1, 2). The cranio-medial quadrants showed a higher PVF than the cranio-lateral quadrants of the front and rear hooves (see Table 1). In contrast, the caudo-lateral quadrants showed a higher PVF than the caudo-medial quadrants of the rear hooves (Table 1). The caudo-lateral quadrants showed a higher PVF than the cranio-lateral quadrants of the rear hooves. No specific pattern was found for the VI of the cranio-lateral and caudo-lateral quadrants of the rear hooves (Vertical pressure distribution and comparisons between different quadrants of the same hoof are shown in Supplementary Materials).

**Figure 3:**
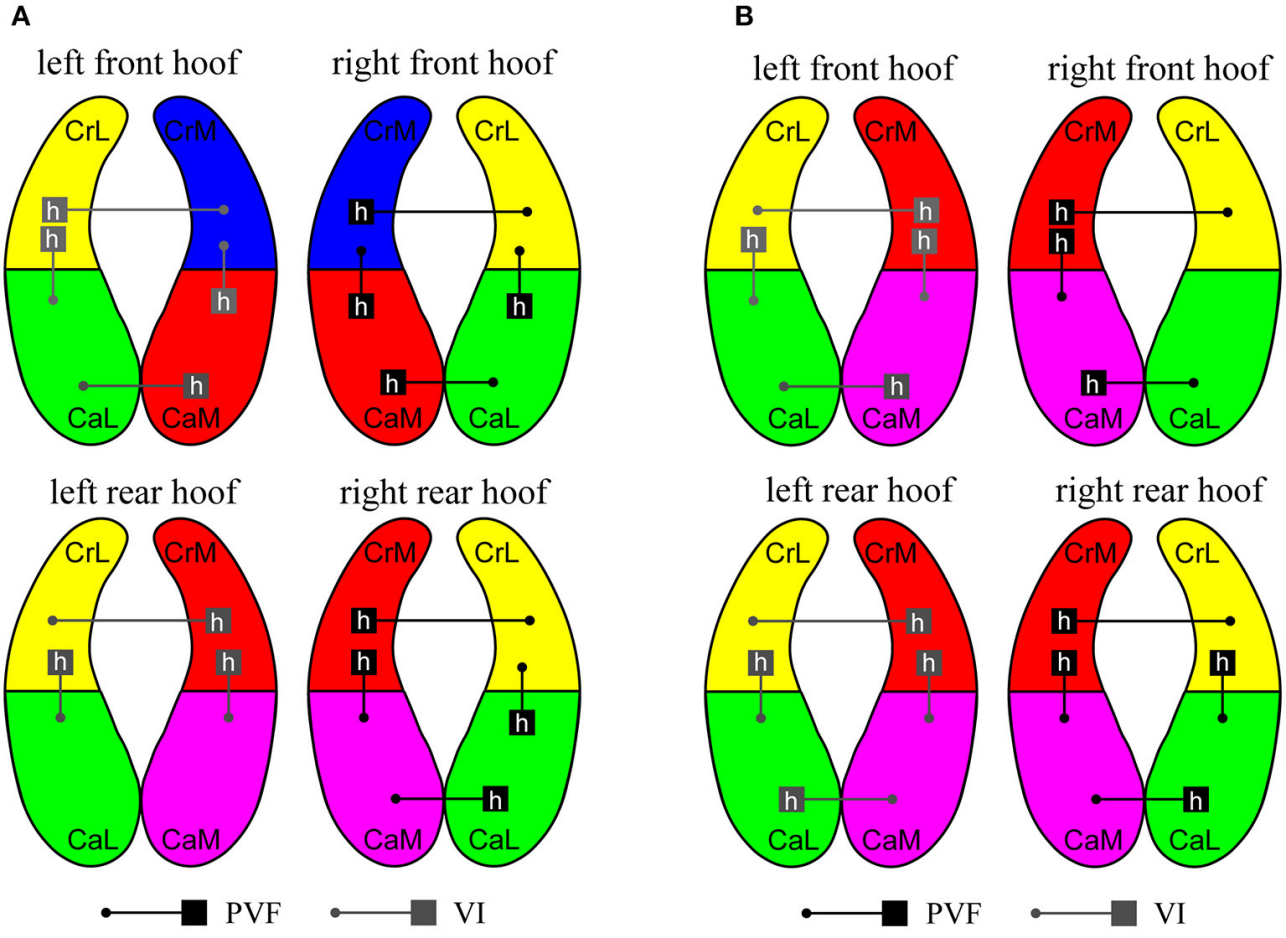
When the slope is 0~25 degrees, PVF and vertical impulse (VI) of the quadrants in each hoof. **(A)** The slope is 0~15 degrees. **(B)** The slope is 20~25 degrees. Each hoof is divided into four quadrants: cranio-medial (CrM), cranio-lateral (CrL), caudo-medial (CaM), and caudo-lateral (CaL). According to the data in Tables 1, 2, the difference between the quadrants in the hoof and the left hoof is similar to that in the right hoof. Therefore, the gray line on the left hoof represents the difference in VI, while the black line on the right hoof represents the difference in PVF. The higher values in the quadrants being compared are denoted by h. In our study, the quadrants with higher values of PVF and VI are defined as the quadrants that suffer more force (the red quadrant in the figure).

The results of the present study indicate that when walking on flat ground, the caudal quadrants of the front hoof were exposed to higher vertical forces than the cranial quadrants, and the cranial quadrant of the medial quadrant of the rear hoof was exposed to higher vertical forces than the caudal quadrant (see Figure 3A). This result is different from those of previous studies in dogs, which indicated that for both the front and rear paw, the force on the cranial quadrants was higher than that on the caudal quadrants (6, 13, 17).

**Figure 4:**
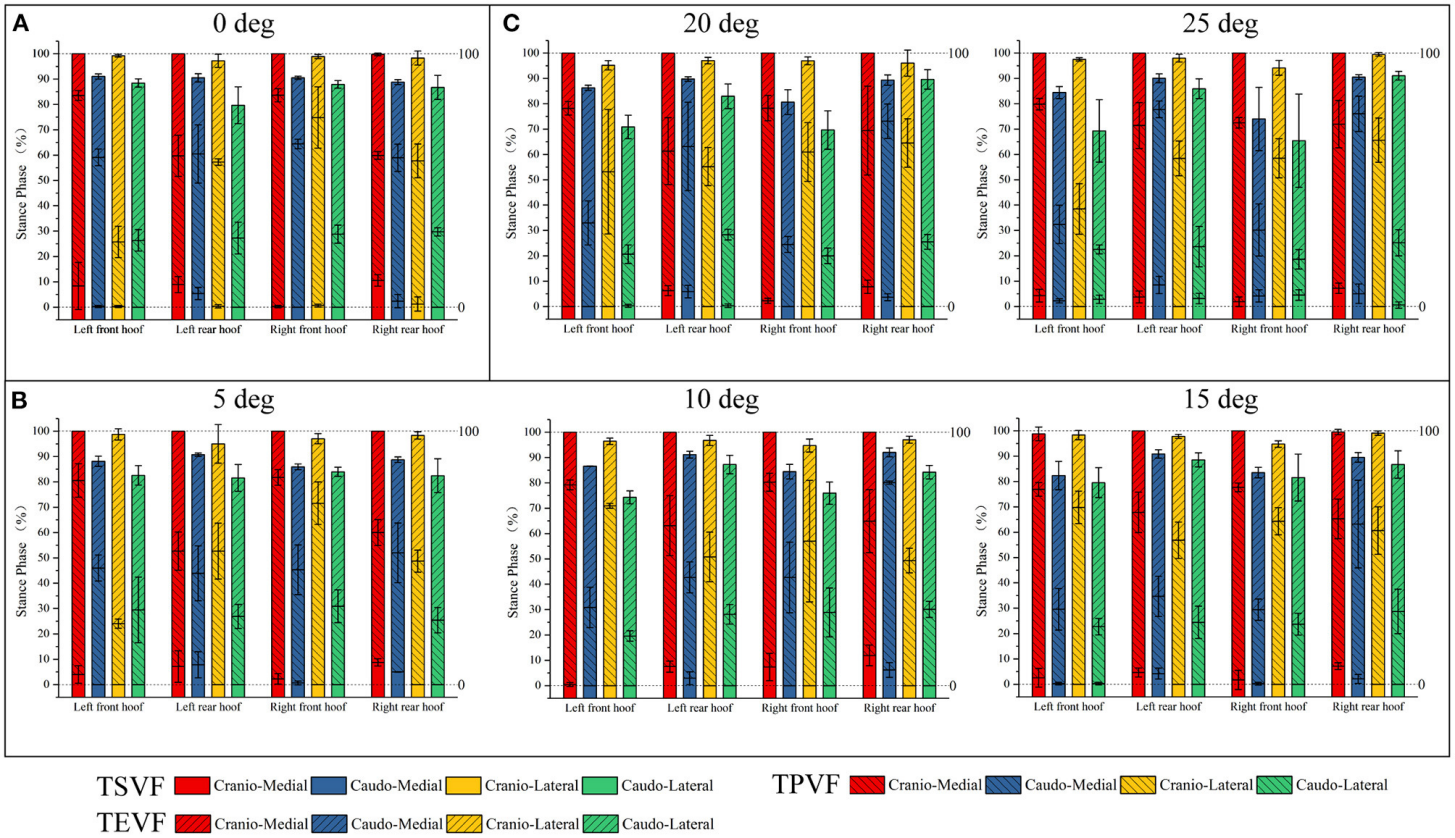
Statistical graphs of TSVF, TPVF and TEVF in different quadrants. **(A)** The slope is 0 degrees. **(B)** The slope is 5, 10, and 15 degrees. **(C)** The slope is 20 and 25 degrees.

For the front and rear hooves, with the exception of the left rear medial quadrants, the TPVF was reached earlier in the caudal quadrants than in the cranial quadrants (see Table 3 and Figure 4A). In contrast, the TPVF was reached earlier in the lateral quadrants than in the medial quadrants (Table 3 and Figure 4A). The TSVF and TEVF were reached earlier in the caudal quadrants than in the cranial quadrants, and in the lateral quadrants than in the medial quadrants. The TSVF and TEVF were reached in caudo-lateral quadrant was reached first and last in the cranio-medial quadrant.

**Table 3:** Values of the time of occurrence of PVF as a percentage of the stance phase duration (TPVF).

**Gradient**		**Cranio-Medial**	**Caudo-Medial**	**Cranio-Lateral**	**Caudo-Lateral**
0	RF	83.63 ± 2.62^1,2,a^	64.43 ± 1.87^1,3^	74.84 ± 12.12^2,4,c,d^	28.79 ± 3.55^3,4^
	RR	59.88 ± 1.49^a^	58.97 ± 5.37^5^	57.77 ± 6.65^6,c^	29.71 ± 1.62^5,6^
	LF	83.53 ± 1.93^7,8,b^	59.09 ± 3.31^7,9^	27.73 ± 6.18^8,d,e^	26.38 ± 4.26^9^
	LR	59.74 ± 8.1^b^	60.48 ± 11.47^10^	57.28 ± 1.31^11,e^	27.26 ± 6.24^10,11^
5	RF	81.74 ± 3.05^1,a^	45.29 ± 9.82^1,2^	71.59 ± 8.38^3,c,d^	30.91 ± 6.46^2,3^
	RR	60.02 ± 5.11^a^	52.02 ± 11.78^4^	48.74 ± 4.34^5,c^	25.40 ± 5.02^4,5^
	LF	80.53 ± 6.59^6,7,b^	46.02 ± 5.14^6,8^	24.07 ± 1.82^7,d,e^	29.50 ± 12.95^8^
	LR	52.94 ± 7.55^b^	43.94 ± 10.84^9^	52.67 ± 11.03^10,e^	26.88 ± 4.74^9,10^
10	RF	80.26 ± 3.57^1,2,a^	42.66 ± 13.92^1,c^	57.01 ± 24.00^2,3^	28.88 ± 9.64^3^
	RR	64.91 ± 12.40^4,a^	80.14 ± 0.64^5,c^	49.37 ± 4.87^4,6^	30.08 ± 3.21^5,6^
	LF	79.21 ± 1.91^7,b^	30.81 ± 7.97^7,8^	70.97 ± 0.99^9^	19.54 ± 2.05^8,9^
	LR	63.11 ± 11.83^10,b^	42.69 ± 6.11^10,11^	50.76 ± 9.79^12^	28.13 ± 3.88^11,12^
15	RF	77.71 ± 1.71^1,2,a^	29.43 ± 4.20^1,3,c^	64.33 ± 5.35^2,4^	23.71 ± 4.31^3,4^
	RR	65.28 ± 7.82^a^	62.23 ± 17.28^5,c,d^	60.66 ± 9.40^6^	28.74 ± 8.75^5,6^
	LF	76.88 ± 2.70^7,b^	29.60 ± 8.20^7^	69.81 ± 6.41^8,e^	22.71 ± 3.22^8^
	LR	67.80 ± 7.97^9,10,b^	34.68 ± 7.96^9,d^	56.82 ± 7.22^10,11,e^	24.44 ± 6.38^11^
20	RF	78.27 ± 4.98^1,2^	24.45 ± 3.15^1,b^	60.96 ± 11.60^2,3^	19.98 ± 3.07^3,d^
	RR	69.41 ± 17.55	73.14 ± 6.77^4,b^	64.52 ± 9.57^5^	25.48 ± 2.89^4,5,d^
	LF	78.22 ± 2.66^6,7,a^	32.99 ± 8.71^6,c^	53.18 ± 24.58^7,8^	20.62 ± 3.66^8,e^
	LR	61.31 ± 13.23^a^	63.16 ± 17.42^9,c^	55.21 ± 7.52^10^	28.34 ± 2.10^9,10,e^
25	RF	72.48 ± 2.06^1,2^	30.17 ± 10.27^1,3,a^	58.51 ± 7.72^2,4,c^	18.62 ± 3.86^3,4^
	RR	71.95 ± 9.36	76.03 ± 6.95^5,a^	65.64 ± 8.72^6^	25.14 ± 5.17^5,6^
	LF	79.82 ± 2.27^7,8^	32.38 ± 7.57^7,9,b^	38.48 ± 9.98^8,10,c,d^	22.53 ± 1.75^9,10^
	LR	71.42 ± 9.06^11^	77.78 ± 3.28^12,b^	58.46 ± 6.87^11,13,d^	23.61 ± 7.95^12,13^

*RF, right front hoof; RR, right rear hoof; LF, left front hoof; LR, left rear hoof*.

*Mean (n = 5) ± standard deviation. The same letter denotes values in the same column with statistically significant differences (comparison of quadrants in the same hoof), and the same number denotes values in the same row with statistically significant differences (comparison of equal quadrants of each hoof)*.

### Distribution Patterns of Blue Sheep Walking on a Small Gradient

The distribution patterns of the PVF and VI when the subjected walked on the ramp with small slopes of 5, 10, and 15° were the same as those when it was walking on flat ground. In general, the front hooves showed a higher PVF and VI than the rear hooves, with the exception of the cranio-medial quadrants (Tables 1, 2). For each slope angle, the caudo-medial quadrants of the front hooves showed a higher PVF and VI than the cranio-medial quadrants of the front hooves. In contrast, for the rear hooves, the cranio-medial quadrants showed a higher PVF and VI than the caudo-medial quadrants. In the front and rear hooves, the caudo-lateral quadrants showed a higher PVF than the cranio-lateral quadrants. Other than the caudal quadrant of the right front hoof and the cranial quadrant of the left front hoof for a slope of 15°, the medial quadrants showed a higher PVF than the lateral quadrants of the front hooves. The cranio-medial quadrants showed a higher PVF than the cranio-lateral quadrants of the rear hooves. In contrast, the caudo-lateral quadrants showed a higher PVF than the caudo-medial quadrants of the rear hooves. The cranio-medial quadrants showed a higher VI than the cranio-lateral quadrants of the rear hooves, except for the right rear hooves on the 5- and 15-degree slopes.

However, the following differences were observed. No specific pattern was found of the VI of the cranio-lateral and caudo-lateral quadrants of the front hooves. Except for the left rear hoof for a slope of 5°, the cranio-lateral quadrants of the rear hooves showed a higher VI than the caudo-lateral quadrants of the rear hooves (Tables 1, 2). The cranio-lateral quadrants showed a higher VI than the cranio-medial quadrants of the front hooves. In contrast, the caudo-medial quadrants showed a higher VI than the caudo -lateral quadrants of the front hooves (Vertical pressure distribution and comparisons between different quadrants of the same hoof are shown in Supplementary Materials). The above results show that the caudo-medial quadrants of the front hooves were exposed to higher forces than the other quadrants, and the cranio-medial quadrant of the rear hooves were exposed to higher forces than the other quadrants (see Figure 3A).

In general, for the front and rear hooves, the TPVF was reached earlier in the caudal quadrants than in the cranial quadrants, with the exception of the medial quadrants of the right rear hoof on the 10-degree slope (64.91 ± 12.40% vs. 80.14 ± 0.64%; Table 3). In contrast, the TPVF was reached earlier in the lateral quadrant than in the medial quadrant (Table 3 and Figure 4B). Exception of the caudal quadrants of the left front hoof at slopes 5, 10, and 15°, the TSVF was reached earlier in the medial quadrants than in the lateral quadrants. The patterns in the TEVF are consistent with that when walking on flat ground.

### Distribution Patterns of Blue Sheep Walking on a Large Gradient

The PVF and VI size relationships between the front and rear hooves when the subject was walking on large slopes of 20 and 25° are the opposite of those on small slopes of 5, 10, and 15°. The rear hooves showed a higher VI than the front hooves, in general, with the exception of the caudal quadrants of the left hooves on the 20-degree slope (Table 2). The rear hooves showed a higher PVF than the front hooves, except for the cranial quadrants of the right hooves and caudal quadrants of the left hooves on the 20-degree slope and the cranial quadrants of the right hooves and caudal-medial quadrants of the left hooves on the 25-degree slope. In general, the rear hooves showed a higher PVF and VI than the front hooves. Details on these analyses are presented in Tables 1, 2.

The distribution patterns similar to walking on a small slope are as follows. For the rear hooves, the cranio-medial quadrants showed a higher PVF and VI than the caudo-medial quadrants (Tables 1, 2), and the cranio-lateral quadrants showed a higher VI than the caudo-lateral quadrants. The cranio-medial quadrants showed a higher PVF than the cranio-lateral quadrants of the rear hooves; in contrast, caudo-lateral quadrants showed a higher PVF than the caudo-medial quadrants. No specific pattern was found in the PVF of the cranio-lateral and caudo-lateral quadrants of the front and rear hooves.

However, the following differences were observed. For the front hoof, the cranio-medial quadrants showed a higher PVF and VI than the caudo-medial quadrants, except for the PVF of the left front hoof on the 20-degree slope. The results were reversed when walking on a small slope. The cranio-lateral quadrants showed a higher VI than the caudo-lateral quadrants of the front hooves. The medial quadrants showed a higher PVF and VI than the lateral quadrants of front hooves, in general, with the exception of the PVF of the caudo-medial quadrants of the right front hoof on the 20-degree slope (Vertical pressure distribution and comparisons between different quadrants of the same hoof are shown in Supplementary Materials). The above results show that the cranio-medial quadrants of the front and rear hooves were exposed to higher forces than the other quadrants (see Figure 3B).

For the front and rear hooves, the TPVF was reached earlier in the caudo-lateral quadrants than in the caudo-medial and cranio-lateral quadrants; in contrast, the TPVF was reached earlier in the cranio-lateral than in the cranio-medial quadrants (Table 3 and Figure 4C). The TSVF was reached earlier in the cranio-lateral quadrants than in the other quadrants. The TEVF was reached at the same time in the caudo-lateral and caudo-medial quadrants, and the patterns in the other quadrants were the same as those when walking on a flat surface.

## Discussion

Previous studies have found that blue sheep have excellent climbing abilities. As the only part of the blue sheep coming into contact with the ground/rocks, sheep hooves play an important role in the climbing process. The VFD of quadruped hooves can be affected by lower limb musculoskeletal diseases (16). Therefore, the force of the blue sheep's hooves and the ground and the transfer pattern of the force between the sheep's hooves can help better determine whether the lower limbs suffer from musculoskeletal diseases. This study quantitatively evaluated the VFD of a healthy blue sheep during climbing on a ramp with different slopes. Based on the obtained results, it was found that the VFD and PVF are affected by slope changes. Therefore, the results of this study provide a new reference for the diagnosis of lower limb musculoskeletal diseases in blue sheep.

The hoof prints of the blue sheep subject were divided into four quadrants, and the PVF, VI, and TPVF of the VFD of each quadrant were compared during walking on the ramp with different slope angles. This approach is different from previous work (3). In a previous study, the sheep's hoof was treated as a whole to compare and analyze the vertical force. Previous studies on dog walking (16) and cat jumping (2) have shown that quadrant division of paw prints can better analyze intra- and inter-investigator variability. These differences could benefit future early diagnosis of orthopedic diseases. For example, a pressure plate can detect weight shifting to reduce pain that occurs when a dog is injured to reduce pain (24). The results of the present study indicate that when blue sheep walk on flat ground, the front hooves bear more vertical force than the rear hooves. This finding is consistent with previous research that considered sheep hooves as a whole (3). In addition, it is consistent with the VFD results of dogs and cats (2, 13), as it is related to the body structure of blue sheep. The body structure of the blue sheep causes the center of gravity to be located on the front side of the geometric center of the torso, which results in the forelimbs needing to bear more weight than the hindlimbs.

The results of the present study indicate that when walking on flat ground, the TPVF occurred earlier in the caudal quadrants than in the cranial quadrants and earlier in the lateral quadrants than in the medial quadrants (see Figure 4A). The quadrants that mainly bears vertical force changed from the caudo-lateral quadrant to the cranio-medial quadrants. A similar finding was reported for cats and dogs, with the main weight transfer occurring from the caudal toward the cranial (cranio-medial) part of the paws (2, 13). Comparisons with cats and dogs can likely provide interspecies insights, especially considering the previous findings of species-related differences in ground reactivity (25). Through a comparison with the previous results, it is found that the vertical force shift of quadrupeds such as blue sheep and cats and dogs in the hoof or paw are consistent.

Blue sheep walk on a wide range of slopes, and the cranio-medial quadrant of the rear hoof is exposed to higher forces than the other quadrants. As the slope increases, the main bearing quadrant of the front hooves changes from the caudo-medial quadrant to the quadrant cranio-medial quadrant. The above results indicate that the VFD of the front hooves of blue sheep is more susceptible to the influence of slope changes than of the rear hooves.

The results show that when blue sheep walk on a slope between 5 and 25°, the proportion of the quadrant where the PVF and VI of the forelimbs are larger than those of the hind limbs decreases gradually with increasing slope. This finding shows that the increase in slope results in the transfer of the main weight of the sheep from the front hooves to the rear hooves.

This study also shows that when blue sheep walk on a slope between approximately 5 and 25° (excluding slopes between ~20° and ~25° medial to the rear hooves and 5° lateral to the left front hoof), the pattern in the TPVF in each quadrant is the same as that on flat ground. This result indicates that the occurrence of the TPVF is not affected by slope changes.

## Conclusions

The study demonstrates that the main stressed quadrant of the front hoof is transferred with increasing slope from the caudo-medial quadrant to the cranio-medial quadrant. The main stressed quadrant of the rear hoof is the cranio-medial quadrant and does not shift with slope changes. When blue sheep walk on any slope, the vertical force shifts from the lateral quadrant to the medial quadrant and from the caudal quadrant to the cranial quadrant. The vertical force are asymmetrical between the front and back hoof. These data may help identify musculoskeletal diseases (such as lameness and osteoarthritis) in blue sheep.

## Data Availability Statement

The original contributions presented in the study are included in the article/Supplementary Material, further inquiries can be directed to the corresponding authors.

## Ethics Statement

The animal study was reviewed and approved by Changchun Zoological and Botanical Garden. Written informed consent was obtained from the owners for the participation of their animals in this study.

## Author Contributions

HK and XL were responsible for experiments and manuscripts preparation. ZQ and LR worked as supervisor for all procedures. All authors contributed to the article and approved the submitted version.

## Conflict of Interest

The authors declare that the research was conducted in the absence of any commercial or financial relationships that could be construed as a potential conflict of interest.
