# Supplementary material for: Hoof Pressure Distribution Pattern of Blue Sheep During Walking on Different Slopes: A Subject-Specific Analysis
Source: Front Vet Sci. 2021 Apr 9;8:633509. doi: 10.3389/fvets.2021.633509 (PMC8062745; doi:10.3389/fvets.2021.633509)
Supplement: Supplementary file 1 [file Data_Sheet_1.PDF]

Supplementary Material

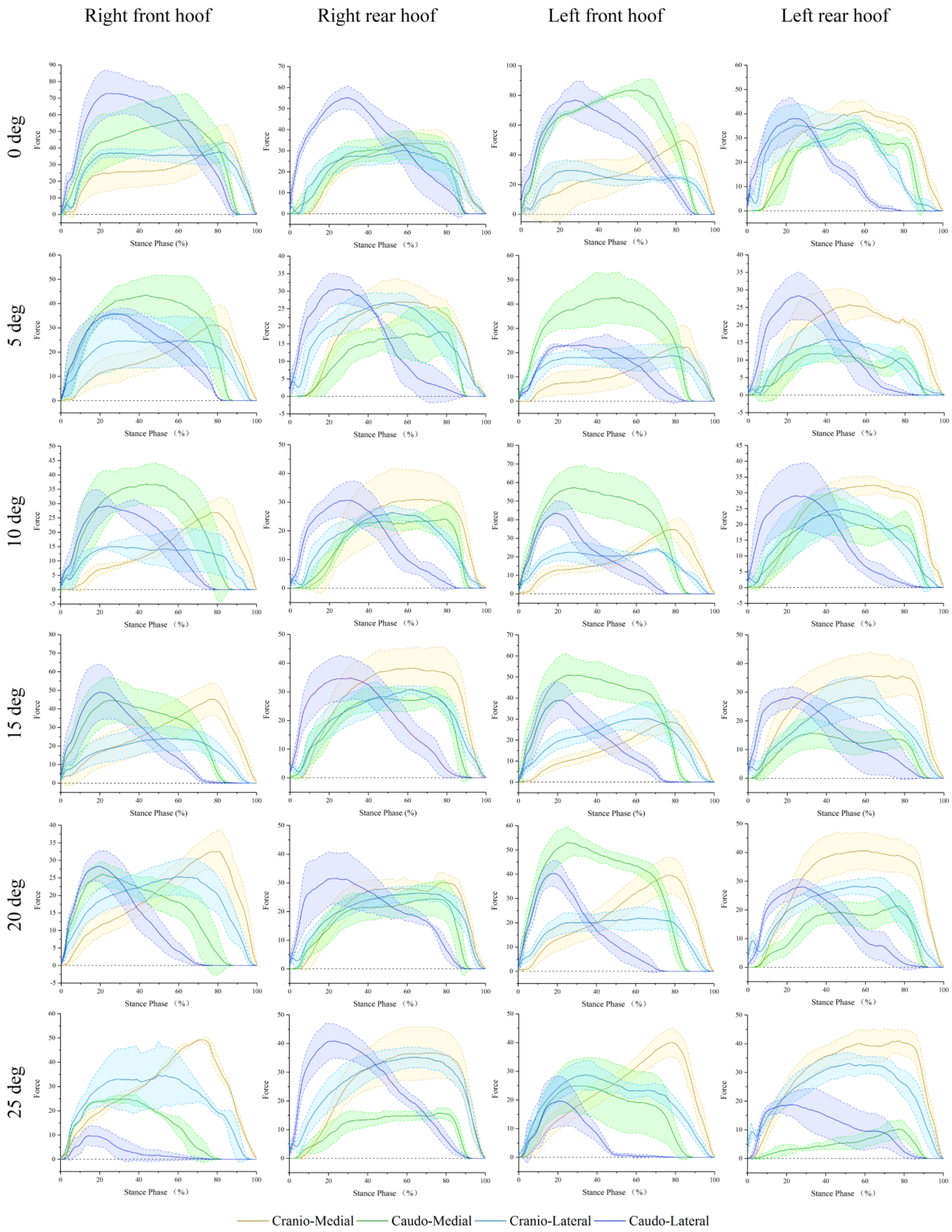

Fig. 1 Force time curve for each quadrant

Fig. 2 shows the vertical pressure distribution for all slopes. The pressure distribution is colour-coded from blue (low pressure) to red (high pressure).

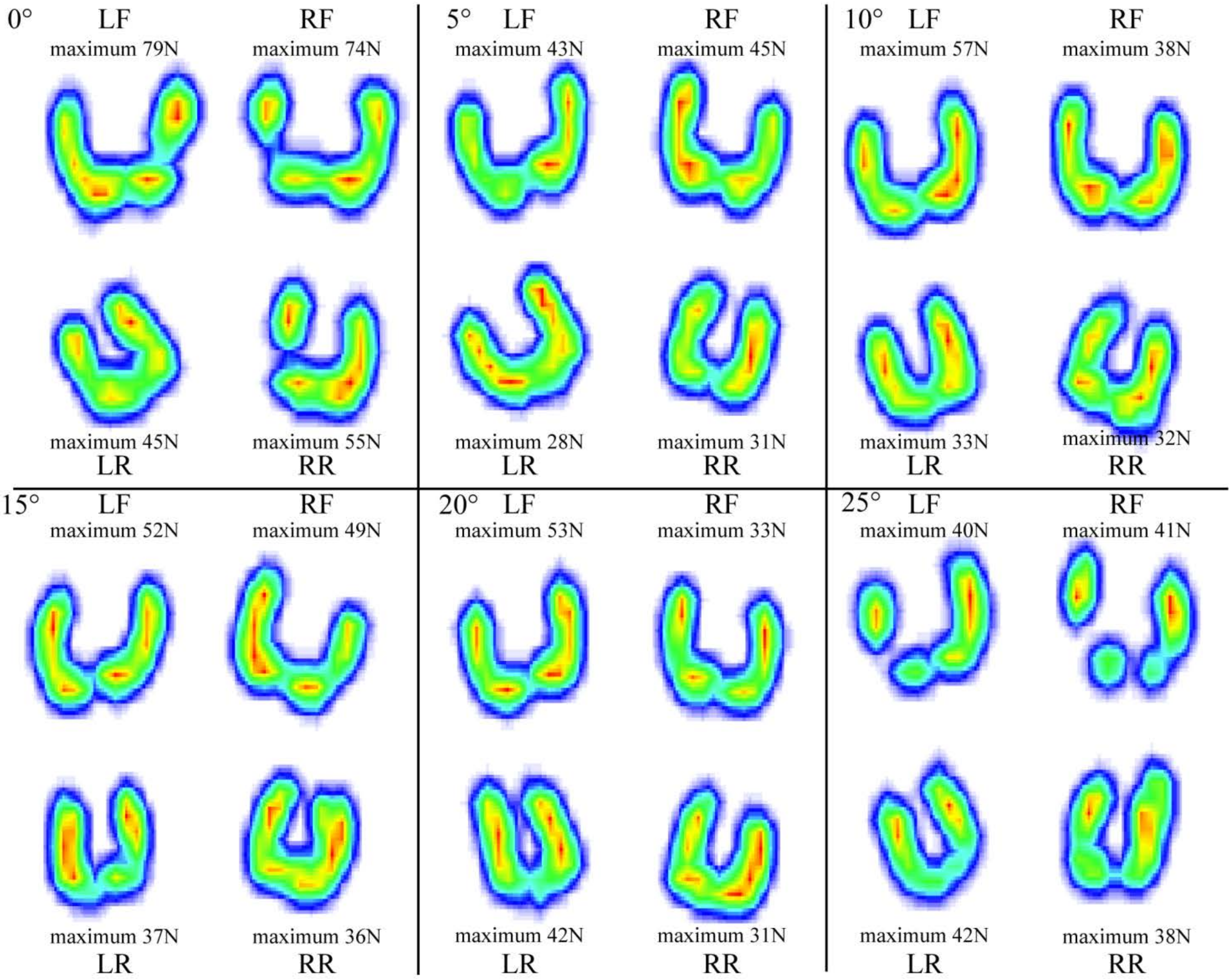

Fig.2 Vertical pressure distribution of the hooves at all slopes

In Fig. 3, the rectangle represents the quadrant, which filled with heavy black represents the right hoof, and filled with light black represents the left hoof. The PVF or VI of the black filled rectangle is larger than that of the unfilled one. As shown in Fig. 2, the PVF in the cranio-medial quadrant of the front hoof is larger than that in the caudo-medial quadrant of the front hoof (excluding the left forehoof at 20°) at 20° and 25°, which is opposite of 0° ~ 15°. At 25°, the PVF in the cranio-lateral quadrant is larger than that in the caudo-lateral quadrant, contrary to 0~20°. At 20°~25°, the PVF in the cranio-lateral quadrant of the hind hoof is bigger than that in the cranio-medial quadrant, which was just opposite from 0°~15°. These results indicate that the PVF of the front foot shifted from the caudal quadrant to the cranial quadrant with increasing slope, and the PVF of the rear hoof shifted from the caudo-medial quadrant to the cranio-medial quadrant with the increase of slope.

As shown in Fig. 2, the VI in the cranio-medial quadrant of the forehoof at 20° and 25° is bigger than that in the caudo-medial quadrant (excluding the left forehoof at 20°). It is just the opposite of 0° ~ 15°. At 20 and 25°, the VI in the cranio-lateral quadrant of the front hoof is larger than that in the

caudo-lateral quadrant, contrary to 0°. At 20°~25°, the VI in the cranio-medial quadrant of the front hoof is larger than that in the cranio-lateral quadrant, contrary to that in 5°~15°.These results show that the VI of the front foot shifted from the caudal quadrant to the cranial quadrant and from the cranio-lateral quadrant to the cranio-medial quadrant with the increase of slope.

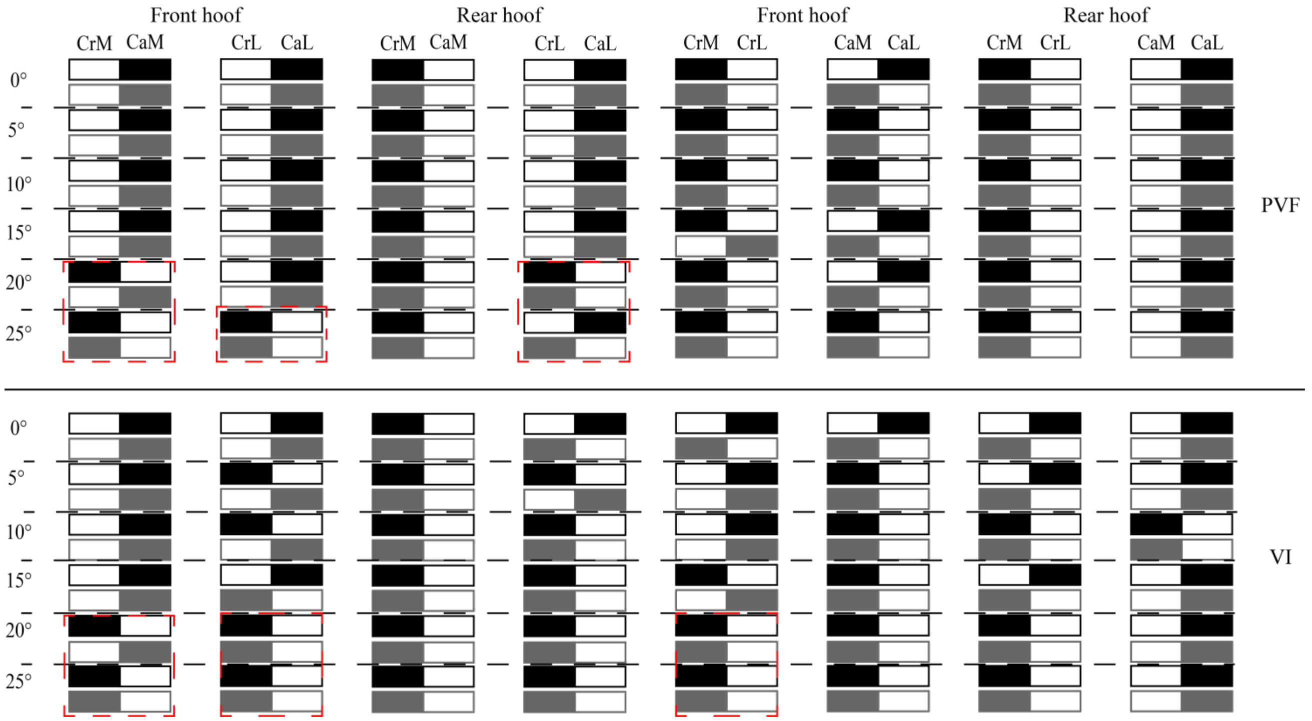

Fig.3 Comparison of PVF and VI in different quadrants of the same hoof

Each hoof is divided into four quadrants: cranio-medial (CrM), cranio-lateral (CrL), caudo-medial (CaM), and caudo-lateral (CaL).

frontiers
